# Localization and Absolute Quantification of Dopamine in Discrete Intravesicular Compartments Using NanoSIMS Imaging

**DOI:** 10.3390/ijms23010160

**Published:** 2021-12-23

**Authors:** Stefania Rabasco, Tho D. K. Nguyen, Chaoyi Gu, Michael E. Kurczy, Nhu T. N. Phan, Andrew G. Ewing

**Affiliations:** 1 Department of Chemistry and Molecular Biology, University of Gothenburg, SE-412 96 Gothenburg, Sweden; stefania.rabasco@gu.se (S.R.); duc.khanh.tho.nguyen@gu.se (T.D.K.N.); chaoyi.gu@chem.gu.se (C.G.); nhu.phan@chem.gu.se (N.T.N.P.); 2 Drug Metabolism and Pharmacokinetics, Research and Early Development, Cardiovascular, Renal and Metabolism (CVRM), BioPharmaceuticals R&D, AstraZeneca, SE-431 83 Mölndal, Sweden; michael.kurczy@astrazeneca.com

**Keywords:** NanoSIMS, dense core vesicles, vesicular compartmentalization, dopamine, absolute quantification

## Abstract

The absolute concentration and the compartmentalization of analytes in cells and organelles are crucial parameters in the development of drugs and drug delivery systems, as well as in the fundamental understanding of many cellular processes. Nanoscale secondary ion mass spectrometry (NanoSIMS) imaging is a powerful technique which allows subcellular localization of chemical species with high spatial and mass resolution, and high sensitivity. In this study, we combined NanoSIMS imaging with spatial oversampling with transmission electron microscopy (TEM) imaging to discern the compartments (dense core and halo) of large dense core vesicles in a model cell line used to study exocytosis, and to localize ^13^C dopamine enrichment following 4–6 h of 150 μM ^13^C L-3,4-dihydroxyphenylalanine (L-DOPA) incubation. In addition, the absolute concentrations of ^13^C dopamine in distinct vesicle domains as well as in entire single vesicles were quantified and validated by comparison to electrochemical data. We found concentrations of 87.5 mM, 16.0 mM and 39.5 mM for the dense core, halo and the whole vesicle, respectively. This approach adds to the potential of using combined TEM and NanoSIMS imaging to perform absolute quantification and directly measure the individual contents of nanometer-scale organelles.

## 1. Introduction

Monitoring the difference in drug concentration during an assay and as a final concentration at the site of action is a long-standing challenge in the process of drug development and chemical probe design. In addition, dose prediction is a criterion for estimating therapeutic potential in terms of efficacy and safety [1,2]. Intracellular drug distribution is also an important parameter, and drug delivery targeting specific intracellular compartments can help to achieve better bioavailability and activity of drugs, as well as improved delivery systems [3,4,5]. Thus, development and improvement of analytical methods for both high resolution localization and absolute quantification of chemicals intracellularly are essential [6,7,8].

Exocytosis and endocytosis are two cellular processes which are central to cellular function and communication. Moreover, they can be exploited for drug development strategies such as for nanoparticle and nanocarrier-based drug delivery systems [9,10,11]. During exocytosis, vesicles travel close to the plasma membrane, then dock and subsequently fuse with the membrane to release their contents to the extracellular space, where an adjacent cell can pick them up via receptors and generate a response. This is the general basis of intercellular communication.

Pheochromocytoma (PC12) cells are a dopaminergic cell line commonly employed in the study of cellular communication and intracellular dynamics [12]. They contain two types of secretory vesicles, i.e., small clear vesicles (SVs) and large dense core vesicles (LDCVs). SVs refer to relatively small (around 50 nm in diameter) vesicles containing acetylcholine as neurotransmitters, whereas LDCVs are larger (150–300 nm in diameter) and owe their name to the densely packed protein core (mainly comprised of chromogranins) present within them. The existence of this protein core allows the LDCVs to store a relatively high concentration of neurotransmitters, mainly dopamine in the case of PC12 cells, without causing severe osmotic pressure to the vesicular membrane [13]. Surrounding the dense core, a lucent domain called the halo is found, containing additional vesicular components such as adenosine triphosphate (ATP), Ca^2+^ ions, and H^+^, maintaining an intravesicular pH of ~5.5 [14,15,16]. The intracellular level of dopamine can be increased by incubating cells with a dopamine precursor, L-3,4-dihydroxyphenylalanine (L-DOPA), which is readily converted into dopamine inside the cytoplasm via a biosynthetic pathway [17,18]. The newly synthesized dopamine can then be loaded into LDCVs via vesicular monoamine transporter (VMAT) and stored. The compartmentalization between dense core and halo has previously been observed with electron microscopy imaging [13,14,19]. Transmission electron microscopy (TEM) is a widespread technique which allows imaging of fine cellular ultrastructures with subnanometer resolution [20]. Although it provides high spatial resolution, TEM offers limited chemical information and thus, it is not suitable for comprehensive chemical profiling of complex biological samples.

Nanoscale secondary ion mass spectrometry (NanoSIMS) imaging offers excellent lateral (down to 50 nm) and mass (>10,000) resolution, high sensitivity (ppm to ppb range) as well as information regarding the chemical composition of a sample [21]. In NanoSIMS, a sample surface is rastered by a primary ion beam, typically Cs^+^ or O^−^, in a pixel-by-pixel configuration. Sputtered secondary ions are then collected and transported to a mass spectrometer, where they are separated according to their mass to charge (*m*/*z*) ratios, and finally detected. Images are generated as color maps showing signal intensities across the scanned area for up to 7 selected secondary ions. This technique allows subcellular and nanometer-scale localization of atomic species such as non-native elements and isotopically tagged molecules [21]. The co-axial configuration of primary and secondary ion beams, which is the uniqueness of NanoSIMS, allows the use of lower beam currents. This improves spatial resolution while still preserving the high level of secondary ion collection, which boosts sensitivity.

Traditionally, relative quantification of target molecules is possible using NanoSIMS, whereas methods for absolute quantification have been lacking. Recently, Thomen et al. developed an approach to quantify the absolute concentration of ^13^C-tagged dopamine in LDCVs of PC12 cells following ^13^C L-DOPA incubation [22]. This allows absolute dopamine concentrations to be determined in individual vesicles and the results were close to the values calculated from electrochemistry measurements, with differing degrees of accuracy depending on the NanoSIMS parameters used. They introduced an approach to localize and carry out absolute quantification of chemical species simultaneously within nanoscopic cellular structures. However, imaging of nanoscale subvesicular structures such as the dense core and halo in LDCVs with SIMS remains a challenge due to their relatively small size.

In this paper, we developed a protocol to observe subvesicular compartmentalization with a combination of TEM and NanoSIMS imaging, and applied it to determine the absolute concentrations of dopamine in discrete vesicular compartments as well as in the entire vesicle. To achieve this, the aforementioned approach presented by Thomen et al. [22] for absolute quantification was combined with an oversampling approach to achieve high lateral resolution during NanoSIMS imaging [23]. The results show that dense core and halo of LDCVs in PC12 cells can be discerned in NanoSIMS images, and ^13^C dopamine is preferentially stored in the dense core after 4–6 h of ^13^C-L-DOPA incubation. In addition, the absolute concentrations of ^13^C dopamine in both compartments can be measured separately. This builds upon the possibility of using combined TEM and NanoSIMS imaging for the investigation of absolute concentration of drugs and metabolites at the sub-organelle scale.

## 2. Results and Discussion

### 2.1. Imaging Workflow and NanoSIMS Parameters

PC12 cells were incubated for 4–6 h with 150 μM ^13^C-labelled L-DOPA, then a series steps of chemical fixation, resin embedding, and thin sectioning were performed, and the sectioned cells were eventually placed onto copper grids. They were first imaged with TEM, during which LDCVs were localized and large vesicles (diameter > 100 nm) with clear compartmentalization between dense core and halo were selected as targets for NanoSIMS imaging.

The NanoSIMS was tuned to detect five secondary ion masses, i.e., ^12^C^14^N^−^, ^13^C^14^N^−^, ^12^C_2_^−^, ^13^C^12^C^−^, and ^34^S^−^. The parameters of the primary column were tuned to improve lateral resolution and sensitivity; D1 aperture and L1 were set at 150 μm (D1-4) and 6055.21 V (38,000 units), respectively. D1 is used to control angular aperture and it affects both the current of primary ion beam and the spot size, which in turn affect lateral resolution. L1 is an electrostatic lens which can also be adjusted to improve lateral resolution by further demagnifying the spot size of the primary ion beam. With increased L1 value, the current of primary ion beam first increases, then peaks, and subsequently decreases. Therefore, a high value of L1 can help produce a low-current, highly focused primary ion beam. A primary ion beam with a current of 0.3 pA can achieve a lateral resolution of 50 nm [21]. However, low current can result in low secondary ion yield and thus, low sensitivity. In this study, the resulting primary ion beam current was 0.2 pA, and the local isotopic enrichment was high enough to produce a high yield of secondary ions even at such low current. Therefore, the combination of these parameters concerted a highly focused primary ion beam with a small spot size and high sensitivity.

Prior to measurements, areas of interest were implanted with a fluence of 2·10^16^ Cs^+^·cm^–2^ to increase the signals of secondary ions. A typical dose required to reach the steady state of secondary ion signal is between 5·10^16^ and 1·10^17^ Cs^+^·cm^–2^, and it can vary for different ions. Here, due to the relatively high enrichment of ^13^C (over 2000 per mille in highly enriched regions of interest (ROIs), Table S1), it was sufficient to implant using a lower dose. This can be explained as the uncertainty during transient state between different planes would be negligible compared to the total ^13^C enrichment in the ROIs. For example, with an isotopic enrichment of 2000 per mille and a variation in isotopic ratio of 50 per mille, an error of 2.5% can be calculated which would be acceptable. Since implantation causes the sputtering of the topmost layer of the material and occasionally leads to the erosion of entire vesicles off the sample surface, implanting a reduced dose also minimizes the loss of vesicle material.

In order to push the limit of the lateral resolution of NanoSIMS, an oversampling method was applied. A raster size of 5 × 5 or 7 × 7 μm^2^ with pixel resolution of 512 × 512 was selected and the pixel size was thus 10–13 nm. Assuming a spot size of 50 nm, this means that the images were oversampled. The use of oversampling to improve spatial resolution in mass spectrometry was first introduced by the Sweedler group, who demonstrated the feasibility of having imaging features smaller than the beam size by overlapping laser shots in matrix-assisted laser desorption/ionization mass spectrometry (MALDI-MS) imaging [23]. This approach has since become a common practice in MALDI-MS [24,25,26,27]. In our study, it allowed discerning dense cores and halos within LDCVs. Although this extends imaging time, the use of small spot size and high pixel scale also allows the reduction of beam mixing, which occurs when imaging a sample feature that has a size smaller or close to that of the primary ion beam, the material sputtered from the feature can be diluted by the sputtering of material which is in its immediate surrounding [22]. In this work, this effect was mitigated by improving lateral resolution and selecting vesicles with diameter larger than the size of the primary ion beam.

### 2.2. ^12^C^14^N Compartmentalization in Relation to Distinct Vesicular Subdomains

**Figure 1:**
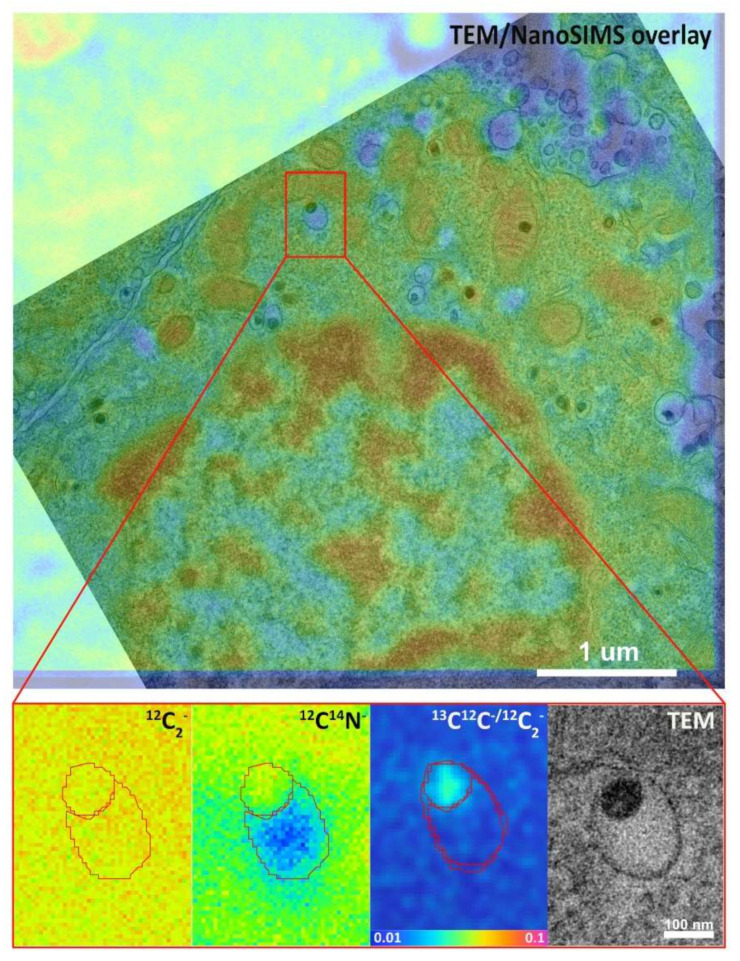
The overlay of ^12^C^14^N^−^ secondary ion image from Nanoscale secondary ion mass spectrometry (NanoSIMS) and the corresponding transmission electron microscopy (TEM) image showing the localization of a large dense core vesicle (LDCV). Above: image of ^12^C^14^N^−^ and TEM overlay (scale bar is 1 μm). Below (from left to right): images of ^12^C_2_^−^, ^12^C^14^N^−^, ^13^C^12^C^−^/^12^C_2_^−^ hue-saturation-intensity (HSI) and TEM of a selected vesicle (scale bar is 100 nm).

**Figure 2:**
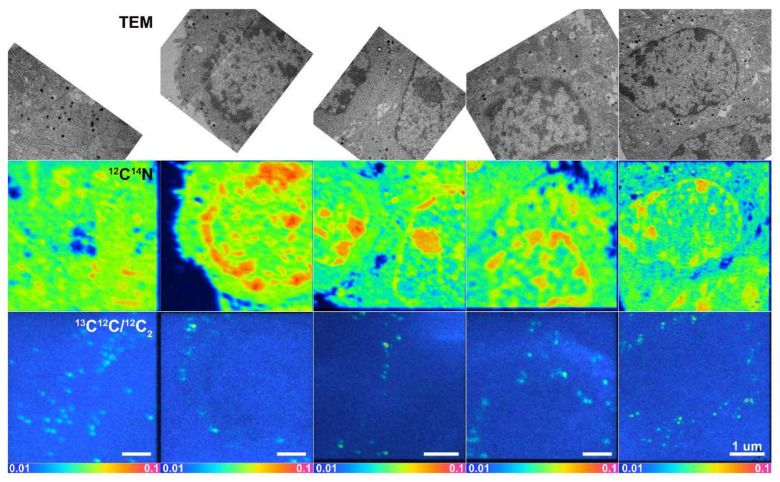
Matched TEM images over NanoSIMS images of 5 different pheochromocytoma (PC12) cells treated with 150 μM ^13^C L-3,4-dihydroxyphenylalanine (L-DOPA) for 4–6 h. From top to bottom: TEM images; NanoSIMS images of ^12^C^14^N^–^ secondary ion species; and HSI images of ^13^C^12^C^−^/^12^C_2_^−^ secondary ion ratio. Scale bar is 1 μm for all images.

**Figure 3:**
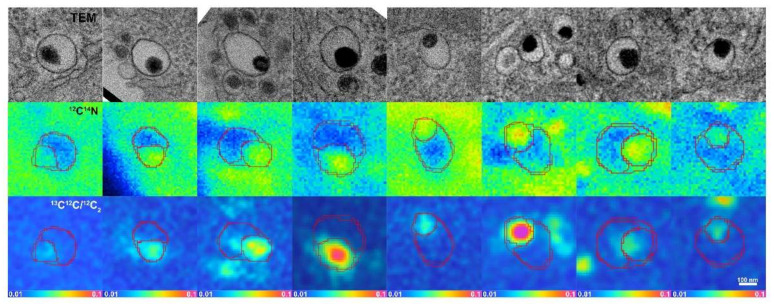
Images demonstrating the compartmentalization between dense core and halo in eight different LDCVs. Top: TEM images of the eight vesicles presenting halo and dense core compartmentalization; middle: ^12^C^14^N^−^ secondary ion images of the eight vesicles obtained from NanoSIMS; bottom: ^13^C^12^C^−^/^12^C_2_^−^ HSI images. Scale bar is 100 nm.

NanoSIMS images were dead time-corrected, drift-corrected and accumulated. TEM images were overlaid with the corresponding ^12^C^14^N^−^ secondary ion images from NanoSIMS, which allowed us to determine the location of LDCVs in the NanoSIMS images. An example of this is shown in Figure 1. An additional overlay of hue-saturation-intensity (HSI) images of ^13^C^12^C^−^/^12^C_2_^−^ secondary ion ratios reveals the localization of the ^13^C enrichment. The HSI model is a transformation of the red, green and blue (RGB) model. It codes the ratio value and statistics for the number of ions detected in each pixel, creating a color scheme which helps the human eyes to recognize ROIs more readily [28]. TEM images and corresponding NanoSIMS images are shown in Figure 2. As mentioned above, LDCVs are easily recognized in TEM images owning to their characteristic dense core structure. However, from a ^12^C^14^N NanoSIMS image alone, is it not possible to distinguish them from a variety of other cellular organelles and cytoplasmic features where the amount of ^12^C^14^N inherently differs. Here, by overlaying TEM and NanoSIMS images, it is possible to localize LDCVs in the secondary ion images. Owing to the high lateral resolution of the NanoSIMS, different vesicle compartments can be distinguished based on their distinct ^12^C^14^N content; namely, the dense core is present as a ^12^C^14^N rich domain due to the densely packed protein, while the halo appears to be ^12^C^14^N depleted. In total, eight vesicles displayed this pattern and these are shown in Figure 3.

### 2.3. Dopamine Concentration: Dense Core versus Halo

ROIs were drawn for each vesicle, delineating perimeters for dense core, halo, and the whole vesicle. Isotopic carbon enrichment (δ^13^C) in per mille (‰) was calculated relative to the Vienna Pee Dee Belemnite (VPDB = 0.0112372) standard and being defined as follows [29],

(1) $$\delta^{13}C=\frac{\frac{13_{C}}{12_{C}}}{0.0112372}\;\times \;1000-1000$$

where the value of ^13^C/^12^C is taken as (^13^C^12^C/^12^C_2_)/2 as we are looking at the enrichment of ^13^C in C_2_ secondary ions. Enrichment values in per mille were then converted to concentration values by using the equation developed by Thomen et al. [22]. Briefly, we assume a contribution of biomass, embedding resin, and ^13^C dopamine towards the total resin-embedded cell material, and the contribution of ^13^C-labeled dopamine is converted to concentration with the equation,

[dopamine] = (δ^13^C_ROI_ − δ^13^C_control_) × 0.101 (2)

where [dopamine] is the concentration of ^13^C dopamine in mM in an ROI, δ^13^C_ROI_ is the enrichment of ^13^C in per mille within the ROI, δ^13^C_control_ is the ^13^C enrichment in per mille in a non-enriched ROI within the cell and is considered as the background, and 0.101 is the factor calculated by dividing the concentration of ^13^C in the resin-embedded cell material over the number of ^13^C atoms in the isotopically labelled ^13^C_6_-dopamine.

**Figure 4:**
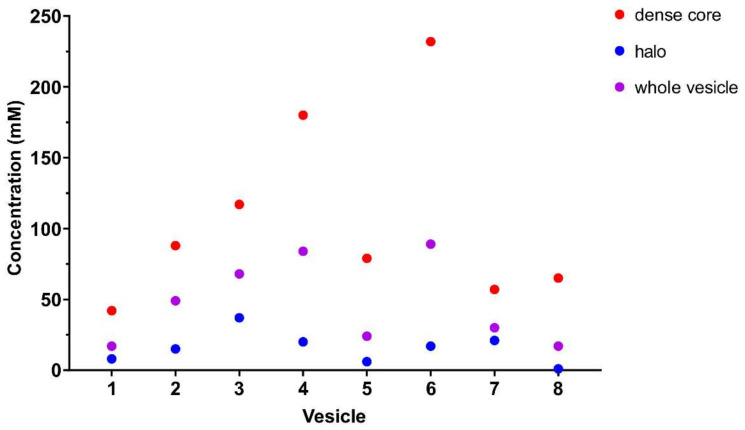
Dot plot showing the concentrations of ^13^C dopamine in dense core, halo and the whole vesicle for eight different vesicles after 150 μM 4–6 h ^13^C L-DOPA treatment.

Concentration values for each ROI and vesicle are shown in Figure 4 and listed in Table S1. Concentrations for the dense core ROIs (red) are higher than those for the halo ROIs (blue), indicating that the enrichment of ^13^C dopamine is mostly localized in the dense core. This demonstrates that the ^13^C dopamine being synthesized during 4–6 h after ^13^C L-DOPA incubation is preferentially stored in the dense core, which is consistent with previous reports of compartmentalized storage of dopamine within LDCVs [22,30].

**Figure 5:**
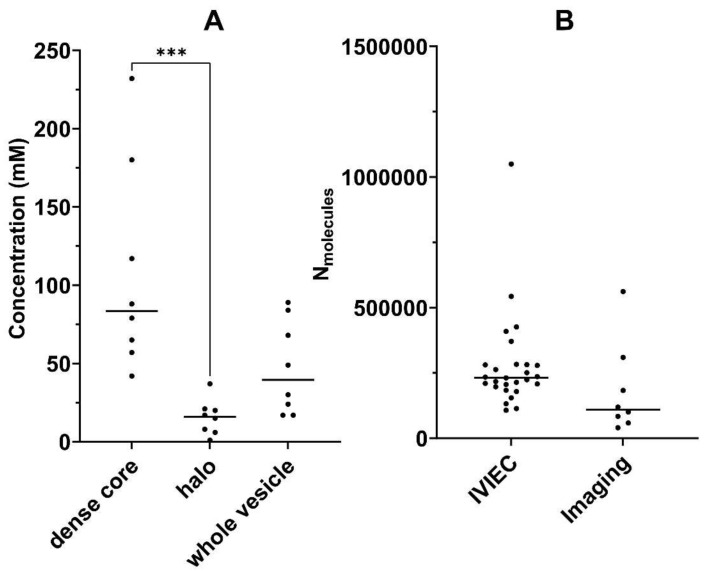
Comparison of ^13^C dopamine concentrations in different compartments of vesicles, and the number of dopamine molecules calculated from intracellular vesicle impact electrochemical cytometry (IVIEC) and imaging. (**A**) Dot plot of ^13^C dopamine concentrations in dense core, halo and the whole vesicle for eight different vesicles, *** *p* < 0.0002, and (**B**) dot plot of the average number of dopamine molecules stored in single vesicles measured with IVIEC (*n* = 27) and imaging (*n* = 8). PC12 cells were pre-treated with ^13^C L-DOPA (150 μM, 4–6 h) and L-DOPA (150 μM, 5 h) for imaging and IVIEC, respectively.

Figure 5A shows grouped concentrations (eight vesicles in the group) for the dense core, halo and the whole vesicle ROIs, and median values are displayed. The median concentrations are 87.5 mM, 16.0 mM and 39.5 mM for dense core, halo and the whole vesicle ROIs, respectively. The data ranges are attributed to biological variations among different vesicles as well as deviations from hand-drawn ROIs. The range of ^13^C dopamine concentration values calculated when using the whole vesicle ROIs is 17–89 mM. These values are consistent with reported values obtained with electrochemical methods (approximately 60–100 mM) [13,31] but fall on the lower end of the range. This might be explained by the fact that the electrochemical technique measures the entire dopamine content of a vesicle (the original dopamine content stored in the vesicle plus the dopamine being newly loaded during L-DOPA incubation), whereas NanoSIMS imaging can only detect the portion of dopamine that is labelled with ^13^C originated from ^13^C L-DOPA incubation, which is only part of the total dopamine in the vesicle. It is important to note that we can measure concentration changes, but not total concentration.

### 2.4. Total Dopamine Concentration: Electrochemistry versus Imaging

Intracellular vesicle impact electrochemical cytometry (IVIEC, details are included in Section 3), an electrochemistry technique that can be used to quantify the total number of dopamine molecules stored inside individual vesicles, was performed to validate vesicular loading [32,33,34]. The results show that the average number of dopamine molecules stored per vesicle increases proportionally as the incubation time of L-DOPA increases (from 1 to 11 h) (Figure S1). Since increased incubation time also leads to increased vesicle volume, the number of dopamine molecules per vesicle increases to maintain a stable intravesicular transmitter concentration [13]. The concentration is calculated by [31],

(3) $$C=\frac{N_{molecules}}{\mathrm{NA}\;\frac{4}{3}\;\pi \;{\left(\frac{D_{ves}}{2}\right)}^{3}}$$

where C is the vesicular concentration of dopamine in mol/L, *N_molecules_* is the number of dopamine molecules quantified from IVIEC measurement, NA is 6.022 × 10^23^ mol⁻^1^, and *D_ves_* is vesicle diameter in dm.

The number of molecules can be obtained by simply rearranging the equation for *N_molecules_*. Here, the number of molecules was calculated for each of the vesicles of interest using the concentration measured via NanoSIMS and the vesicle diameter measured via TEM. These values were compared with the average number of molecules per vesicle directly quantified by IVIEC in 27 live cells incubated with 150 µM L-DOPA for 5 h. The results are shown in Figure 5B and listed in Table S1.

An unpaired *t*-test was applied to check for significant difference between the mean concentration values of IVIEC and imaging, and this resulted in a *p* value of 0.2005, confirming that there is no statistical difference between the means of the two data sets. This supports the validity of combining imaging data and the Equation (1) to quantify the absolute concentration of ^13^C dopamine in vesicles.

## 3. Materials and Methods

### 3.1. Cell Culture

PC12 cells were received as a gift from Lloyd Greene at Columbia University and cultured as previously described [35]. Briefly, cells were grown in RPMI-1640 medium (Sigma-Aldrich, Stockholm, Sweden) supplemented with 10% horse serum (Sigma-Aldrich, Sweden) and 5% fetal bovine serum (Sigma-Aldrich, Sweden) on mouse type IV collagen-coated T-75 flasks (Corning BioCoat, Fisher Scientific, Gothenburg, Sweden), and subcultured every 7 days. The medium was replaced every 2 days and cells were maintained in an incubator at 37 °C with 7% CO_2_ and 100% humidity during the entire culture. For NanoSIMS experiments, cells were subcultured on 35 mm glass-bottom dishes (MatTek Life Science, Ashland, MA, USA) coated with poly-D-lysine (Sigma-Aldrich, Sweden) and the incubation experiment was performed on the third day after plating. For IVIEC experiments, cells were subcultured on 60 mm type IV collagen-coated dishes (Corning BioCoat, Fisher Scientific, Sweden) and maintained for 4 days until the experiment.

### 3.2. Sample Preparation

^13^C L-DOPA and L-DOPA stock solutions were prepared by dissolving the isotopically labeled ^13^C L-DOPA (1−13C, RING-13C6, 99%, Cambridge Isotope Laboratories Inc., Andover, MA, USA) and L-DOPA (Sigma-Aldrich, Sweden), respectively, in phosphate-buffered saline (Sigma-Aldrich, Sweden) in the dark while purging with argon (6.0, Linde Gas AB, Solna, Sweden). The final concentration required for the incubation was obtained by diluting the stock solution in cell medium. For NanoSIMS experiments, cells were first incubated with 150 μM ^13^C L-DOPA for 4 or 6 h and then fixed in Karnovsky fixative (2.5% glutaraldehyde, 2% formaldehyde, 0.02% sodium azide in 0.05 M sodium cacodylate buffer) for 30 min. Chemicals were purchased from Sigma-Aldrich, Sweden. Next, after washing with 150 mM sodium cacodylate buffer, the cells were kept overnight at 4 °C. Afterwards, postfixation with 1% osmium tetroxide (Agar Scientific Ltd., Stansted, UK) at 4 °C for 30 min was followed by staining with 1% uranyl acetate (Merck, Sigma-Aldrich, Sweden) for 20 min. Then, after dehydration with increasing ethanol dilutions (30%, 50%, 70%, 85%, 95% and 99.5%) the cells were finally embedded in epoxy Agar100 resin (Agar Scientific Ltd., UK). Ultra-thin 100 nm thick sections were cut with ultramicrotome (Leica EM UC6) and placed onto Formvar coated copper finder grids (200 mesh, Sigma-Aldrich, Sweden). For IVIEC experiments, cells were incubated with 150 μM L-DOPA for 1, 3, 5, 7, 9, or 11 h. Before the experiments, cell medium was removed from the culture dishes and the cells were then washed three times with a pre-warmed isotonic solution (composition included 150 mM NaCl, 5 mM KCl, 1.2 mM MgCl_2_, 2 mM CaCl_2_, 5 mM glucose, and 10 mM HEPES, all purchased from Sigma-Aldrich, Sweden; pH was adjusted to 7.4 with 3 M NaOH, purchased from Sigma-Aldrich, Sweden; and the solution was filtered prior to use). The cells were kept in 5 mL isotonic solution and maintained at 37 °C with a heating plate during entire IVIEC experiments.

### 3.3. TEM

Electron microscopy imaging was carried out with a Thermo Scientific™ Talos L120C TEM microscope (Center for Cellular Imaging, Sahlgrenska Academy, University of Gothenburg) operated at 120 keV.

### 3.4. Electrode Fabrication and IVIEC Measurements

Nanotip electrodes were fabricated and used to perform IVIEC measurements. The procedure of electrode fabrication was described in pervious publication [32]. A 5 µm-diameter carbon fiber was first aspirated into a borosilicate glass capillary (O.D. 1.2 mm, I.D. 0.69 mm, 10 cm length, Sutter Instrument Co., Novato, CA, USA). The capillary was then pulled into two parts using a vertical micropipette puller (model PE-21, Narishige, Inc., Setagaya-ku, Japan). The carbon fiber outside the capillary was subsequently trimmed to around 100 µm length and flame-etched with a butane gas burner (Clas Ohlson, Gothenburg, Sweden) to reach a sharp shape (diameter around 50–100 nm). Afterwards, epoxy (EPO-TEK 301, G A Lindberg ChemTech AB, Mölnlycke, Sweden) was used to seal the electrodes, which were then baked overnight at 100 °C. Right before the IVIEC experiments, electrode testing was performed using cyclic voltammetry in PBS solution containing 100 µM dopamine, and electrodes showing stable steady-state currents were chosen for the experiments. To do IVIEC, an electrode was first placed on top of a single PC12 cell, then slowly pushed through the cell membrane to reach the cytoplasm where vesicles reside. Due to the constant potential applied to the electrode (+700 mV versus an Ag/AgCl reference electrode), vesicles adsorb and rupture on the electrode surface, and the dopamine molecules inside the vesicles can be oxidized by the electrode and directly quantified [32]. All IVIEC experiments were performed on an inverted microscope (IX81, Olympus) in a Faraday cage. An Axopatch 200B potentiostat (Molecular Devices, Sunnyvale, CA, USA) was used to apply the potential, and the signal output was filtered at 2 kHz and digitized at 5 kHz.

### 3.5. NanoSIMS Measurements

The measurements were performed using a 16 keV Cs^+^ beam of ~0.2 pA (D1-4, L1 = 38,000). The entrance slit was at 15 μm width, the aperture slit was at 150 μm width, and the energy slit was fully open. The field of view was 5–7 μm and the pixel size was 512 × 512. A saturation fluence of 2·10^16^ Cs^+^·cm^–2^ was implanted prior to each measurement. For data analysis, images were drift corrected and accumulated using WinImage (Cameca 2008, version 2.0.7). ROIs were drawn in freehand mode by thresholding the features of the vesicles on the ^12^C^14^N^−^ secondary ion images, and the sum of pixel intensities inside the ROIs for the C_2_ pair was computed as counts/s/pixel with dead time correction. TEM and NanoSIMS overlays were created on Affinity Designer 1.10.1 and statistical analysis was performed using GraphPad Prism 9.1.2.

### 3.6. Data Analysis and Statistics for IVIEC Experiments

Igor Pro 6.37 with a script originating from the David Sulzer group at the Columbia University was used to analyze the IVIEC data. A 1 kHz binomial sm filter was used to filter the IVIEC data and then a criteria of five-time standard deviation of the background noise was defined in the software as the threshold for selecting IVIEC data, which was followed by a careful inspection to avoid false positives. To calculate the average number of dopamine molecules quantified by IVIEC, mean of medians from single PC12 cells was used which can help to decrease the impact of extreme values. Statistics were performed in GraphPad Prism 5 and pairs of data sets were compared with a nonparametric, unpaired, two-tailed Mann–Whitney rank-sum test, *** *p* < 0.001.

## 4. Conclusions

High-resolution imaging of subvesicular compartments of LDCVs in PC12 cells was achieved by combining TEM and NanoSIMS imaging, and absolute concentration of ^13^C dopamine in discrete compartments of LDCVs, i.e., dense core and halo, was quantified. NanoSIMS parameters (primary column, sampling rate) and correlative workflow were optimized to achieve high lateral resolution, which allowed the distinction of subvesicular compartments in a single cell. The results demonstrate that ^13^C dopamine is preferentially loaded into the dense core portion of the LDCVs in PC12 cells after a 4–6 h ^13^C L-DOPA incubation. Moreover, the absolute concentrations of ^13^C dopamine in distinct vesicle domains as well as in the entire vesicle were successfully quantified, which are 87.5 mM, 16.0 mM and 39.5 mM for dense core, halo and the whole vesicle, respectively. In its current form, the equation for calculating absolute quantification can only be used for cell material embedded in the same type of resin and enriched by ^13^C_6_-dopamine. Another limitation is the imaging time required for this approach, meaning that to acquire statistically useful amount of data, relatively long NanoSIMS sessions are needed.

Finally, further investigations into the effect of oversampling on lateral resolution of NanoSIMS, the dynamics of dopamine compartmentalization, and the effects of imaging parameters on absolute quantification will lead to improvement in the current approach, and meanwhile promote the use of imaging for accurate localization and absolute quantification of chemicals at the sub organelle nanoscale.

## Data Availability

The data presented in this study are available on request from the corresponding author.

## Supplementary Materials

The following are available online at https://www.mdpi.com/article/10.3390/ijms23010160/s1.
