# Supplementary material for: Localization and Absolute Quantification of Dopamine in Discrete Intravesicular Compartments Using NanoSIMS Imaging
Source: Int J Mol Sci. 2021 Dec 23;23(1):160. doi: 10.3390/ijms23010160 (PMC8745556; doi:10.3390/ijms23010160)
Supplement: Supplementary file 1 [file ijms-23-00160-s001.zip › ijms-1519048-supplementary.pdf]

SUPPORTING INFORMATION

Localization and absolute quantification of dopamine in discrete intravesicular compartments using NanoSIMS imaging

Stefania Rabasco [1], Tho D. K. Nguyen [1], Chaoyi Gu [1], Michael E. Kurczy [2], Nhu T. N. Phan [1], Andrew G. Ewing [1,*]

[1]Department of Chemistry and Molecular Biology, University of Gothenburg, Gothenburg SE-412 96, Sweden
[2]DMPK, Research and Early Development, Cardiovascular, Renal and Metabolism (CVRM), BioPharmaceuticals R&D, AstraZeneca, Gothenburg, Sweden

*Correspondence: andrew.ewing@chem.gu.se

Table of Contents

Figure S1 1
Table S1 2

Supplementary Figure S1

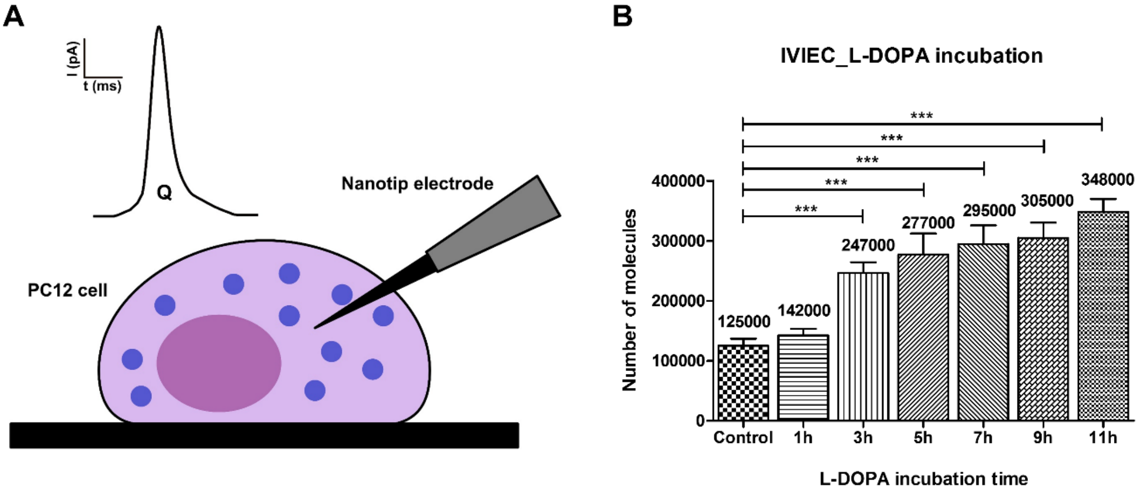

Figure S1. Illustration and results of IVIEC. (A) A scheme showing the IVIEC technique performed with a nanotip electrode. When the electrode is inside the cytoplasm, the oxidation of the dopamine content inside individual vesicles gives rise to amperometric current spikes (an example of the spike is shown). By applying the Faraday equation (N=Q/nF, Q: charge of the spike, n: number of electrons transferred in the dopamine oxidation reaction which is 2, F: Faraday constant), the number of molecules stored in individual vesicles can be calculated. (B) Average number of dopamine molecules stored per PC12 vesicle quantified by IVIEC. The cells were treated with or without 150 µM L-DOPA. For L-DOPA treatment, the incubation times were 1, 3, 5, 7, 9, and 11 h. 11-27 cells were measured for each group. A nonparametric, unpaired, two-tailed Mann-Whitney rank-sum test was applied to compare the L-DOPA treatment groups to the control group, *** $p < 0.001$.

Supplementary Table S1

Vesicle	Dense core $\delta^{13}C_{PDB}(C_2)$ (‰)	Halo $\delta^{13}C_{PDB}(C_2)$ (‰)	Whole vesicle $\delta^{13}C_{PDB}(C_2)$ (‰)	^{13}C dopamine concentration in dense core (mM)	^{13}C dopamine concentration in halo (mM)	^{13}C dopamine concentration in whole vesicle (mM)	Vesicle diameter (nm)	Number of molecules
1	430	95	191	42	8	17	222	$5.86 \cdot 10^4$
2	903	183	516	88	15	49	228	$1.83 \cdot 10^5$
3	1191	401	702	117	37	68	297	$5.62 \cdot 10^5$
4	1771	187	818	180	20	84	227	$3.10 \cdot 10^5$
5	802	85	257	79	6	24	223	$8.39 \cdot 10^4$
6	2339	213	923	232	17	89	162	$1.19 \cdot 10^5$
7	608	254	338	57	21	30	220	$1.01 \cdot 10^5$
8	682	51	213	65	1	17	196	$4.03 \cdot 10^4$

Table S1. Summary of vesicle parameters for the eight vesicles. The parameters include ^{13}C dopamine enrichment values in per mille, ^{13}C dopamine concentration equivalents in mM, vesicle diameters, and number of molecules. Background values are not included.
